# Novel Coronavirus Cough Database: NoCoCoDa

**DOI:** 10.1109/ACCESS.2020.3018028

**Published:** 2020-08-19

**Authors:** Madison Cohen-McFarlane, Rafik Goubran, Frank Knoefel

**Affiliations:** 1 Department of Systems and Computer EngineeringCarleton University 6339 Ottawa ON K1S 5B6 Canada; 2 Bruyére Research Institute Ottawa ON K1R 6M1 Canada; 3 Bruyére Continuing Care 25474 Ottawa ON K1N 5C8 Canada; 4 Elisabeth Bruyére Hospital Ottawa ON K1N 5C8 Canada

**Keywords:** Acoustic signal processing, audio databases, audio systems, biomedical measurement, biomedical monitoring, data analysis, data collection, medical conditions, medical diagnosis, patient monitoring, smart homes

## Abstract

The current pandemic associated with the novel coronavirus (COVID-19) presents a new area of research with its own set of challenges. Creating unobtrusive remote monitoring tools for medical professionals that may aid in diagnosis, monitoring and contact tracing could lead to more efficient and accurate treatments, especially in this time of physical distancing. Audio based sensing methods can address this by measuring the frequency, severity and characteristics of the COVID-19 cough. However, the feasibility of accumulating coughs directly from patients is low in the short term. This article introduces a novel database (NoCoCoDa), which contains COVID-19 cough events obtained through public media interviews with COVID-19 patients, as an interim solution. After manual segmentation of the interviews, a total of 73 individual cough events were extracted and cough phase annotation was performed. Furthermore, the COVID-19 cough is typically dry but can present as a more productive cough in severe cases. Therefore, an investigation of cough sub-type (productive vs. dry) of the NoCoCoDa was performed using methods previously published by our research group. Most of the NoCoCoDa cough events were recorded either during or after a severe period of the disease, which is supported by the fact that 77% of the COVID-19 coughs were classified as productive based on our previous work. The NoCoCoDa is designed to be used for rapid exploration and algorithm development, which can then be applied to more extensive datasets and potentially real time applications. The NoCoCoDa is available for free to the research community upon request.

## I. Introduction

On March 11^th^, 2020 the World Health association (WHO) declared the rapid transmission of the novel coronavirus (COVID-19) a pandemic [1]. Since then researchers around the world have been working to aid in the diagnosis, tracking and treatment of all those affected [2]–[5]. As of June 18^th^, 2020, there have been a total of 10,021, 401 cases worldwide (103,250 Canada) and 499,913 deaths (8,522 Canada) [1], [6]. With so many people affected, novel methods for disease predication and tracking should be considered.

The symptoms of COVID-19 vary considerably depending on the individual and their expression can take up to 14 days to present after an individual is affected [6]. Furthermore, the early symptoms are easily confused with those of the common cold and/or flu (cough, fatigue and fever) [6]. If the virus enters the lungs, it can lead to difficulty breathing, pneumonia and possibly death [6].

Early identification, prior to infection moving to the lungs, would be an important tool in early identification for testing, isolation and contact tracing. Cough signature identification may help differentiate between coughs caused by various viruses, bacteria, or other acute and chronic health conditions. This cough signature could then be used as a screening method at airports, borders and health facility waiting rooms. Furthermore, a monitoring method that harnesses currently available technology would allow for the acquisition of larger amounts of data. As one of the early symptoms of COVID-19 is cough [1], here we introduce the idea of using audio recordings of cough events as an area for further investigation. The long-term goal being the development and implementation of a system that can ideally monitor, detect and classify cough events.

Currently physicians asses cough based on the frequency (how often someone coughs), severity (how forceful the cough event is), and cough characteristics based on known cough types (wet, dry, wheeze, and whooping). The available information to the physician is limited as they tend to rely on clinical observations and self-reporting from the patient. This is exacerbated by the current pandemic with physical distancing and isolation leading to infrequent medical visits, which makes efficient and accurate diagnosis difficult.

Unobtrusive cough assessment methods have a few possible applications. An audio-based assessment tool could be implemented in residential environments as a subsystem of current remote health monitoring systems (e.g. Safety Labs [7]). The same tool could be implemented, with adjustments, to large public spaces like waiting rooms and transit stations. Here, the main goal would be to track individuals who are experiencing coughs associated with a particular disease (e.g. COVID-19) in order to help with contact tracing. Finally, the system could also be implemented in continuing care homes by providing more accurate clinical information about the frequency and severity of cough events to medical professionals.

When considering COVID-19 type cough events, the largest hurdle is the collection of coughs from those testing positive with COVID-19. As of now, the feasibility of a clinical study aimed to record involuntary cough events from COVID-19 positive individuals is unrealistic. This is especially true when considering the time it would take to accumulate this kind of data, not to mention adding more pressure to the healthcare system by adding more work for frontline workers. Additionally, taking a blood sample or an X-ray is much easier than asking frontline workers to get a patient in distress to cough on demand and record it. A clinical study may be possible in the future, but not during an active state of pandemic.

Alternative methods of data collection can be considered in order to start investigating the feasibility of using the audio of coughs as a tool to identify COVID-19. Recently two research groups have focused on asking individuals to record their coughs at home. Cambridge University published an app called *COVID-19 Sounds App* [8], the goal of which is to ask individuals, both healthy and unhealthy (COVID-19 positive), to record their cough and breathing events. They also collect basic demographics, medical history and a voice sample [8]. A group out of L’École Polytechnique Fédérale de Lausanne has created a web-based application, which asks users who have self-reported to have COVID-19 to record cough events using their smartphone [9]. Additional information requested includes any other symptoms experienced by the user [9]. Both applications ask users to cough into the microphone on their device, the recorded coughs would therefore be voluntary in nature. There are key pathophysiological differences between voluntary and reflex cough events [10]. It has also been reported that these two mechanisms are affected differently by disease [10]. As COVID-19 research is in the early days, we don’t know if it affects these types of cough events differently.

Furthermore, there are vast repositories of information available online. Creating tools to harness this pre-existing information to investigate new ideas can be considered an implementation of big data research, which is a growing area of interest [11]. Some areas of big data research include sourcing data from social media [12] and YouTube videos [13]. More recently, researchers have focused on using big data information to evaluate trends in the current COVID-19 pandemic [12], [14], [15].

To our knowledge there are no cough databases currently available that contain reflex COVID-19 cough events. Here we present the Novel Coronavirus Cough Database (NoCoCoDa), which contains recordings of coughs that have been collected through online interviews with COVID-19 positive individuals as an unobtrusive and noncompulsory source of COVID-19 cough events.

## II. Existing Audio Cough Event Databases in the Literature

There are very few databases of audio-based cough events currently available. There are however online databases that include cough events as a class of audio events. This means that the cough events do not necessarily contain additional information (e.g. demographics or disease information). Databases available for use that include cough events are as follows.

### A. Audio Set

Database of audio events from 632 audio classes collected from 10 second segments of YouTube videos. There are 871 cough events and 834 breathing events. There are no further descriptions other than segments being labeled as a cough [16].

### B. DCASE 2016

DCASE 2016 or detection and classification of acoustic scenes and events was a challenge presented in 2016. The second task, sound event detection in synthetic audio, contained 20 cough events [17]. Again, these cough events did not contain any other descriptions or labels.

### C. Freesound

Freesound.org is a free access collaborative collection of sounds [18]. As sounds are collected from many users, recording methods and descriptions vary significantly. When searching for the term ‘cough’, 736 sounds were identified [18], none of which included any other descriptions or labels.

### D. Targeted Cough Research

When looking for a cough specific database, not many are available and even less are free to use. The only one that was found was a collection of cough events compiled from online sources used to evaluate Pertussis [19]. The coughs used were not released as a stand-alone database, however the original links were included as a table in the publication. For completeness, all links were tested and only 23 of the listed 38 were still available. The coughs used in the Pertussis paper included a disease type and age group (infant, child or adult).

Our research group has worked with audio cough events in the past using both a curated collection of cough events [20], [21] and pre-existing databases [22]. The curated collection of cough events were obtained from a sound database [20], personal cough recordings and a subset of cough events included in Smith et. *al.* [23].

## III. Proposed Database: NoCoCoDa

### A. Interview Acquisition

The database of novel coronavirus coughs (NoCoCoDa) contain cough events obtained from online interviews with COVID-19 positive individuals. All interviews were found online and were published by news sources. Searches were conducted from April to June 2020, in order to identify any new interviews. Search terms used are listed in Table 1 and were repeated at least twice on two different days.

**TABLE 1:** Search Terms

Search Term
1. Coronavirus interview
2. Coronavirus infected person interview
3. Coronavirus interview from hospital
4. Coronavirus interview from ICU
5. COVID interview
6. COVID-19 interview
7. Interview with COVID-19 patient

A total of 13 interviews were found involving 10 individuals. Interview information is summarized in Table 2.

**TABLE 2:** COVID-19 Positive Interview Information

Title	Source	Interview Date	Link	Number of Cough Events	Description of Interviewee	Assigned Subject Number
Coronavirus Patient Speaks Out in Exclusive Interview	CBS New York	March 9^th^, 2020	https://www.youtube.com/watch?v=vjJbLJqcVSY	2	Male, no underlying health conditions, COVID-19 positive	S1
COVID-19 patient ‘would beg’ Canadians to obey top doctors, government	CBC News	March 28^th^, 2020	https://www.youtube.com/watch?v=yr2GnZo2KiA	15	Female, 28 during the 10^th^ symptomatic day	S2
DocMom Describes Watching Her Child Battle COVID-19	Now This News	April 22^nd^, 2020	https://www.youtube.com/watch?v=qDkI7lfhjgw	13	Male child (four years old)	S3
Georgia coronavirus patient talks about what it’s like living with COVID-19 AJC	Atlanta Journal-Constitution	March 13^th^, 2020	https://www.youtube.com/watch?v=XXjthKeJqOc	3	Male self-reporting as also having pneumonia and diabetes	S4
He’s Infected. See his message from hospital room	CNN	March 11^th^, 2020	https://www.youtube.com/watch?v=vcb38Pbmmpw	2	Male self-reported as having viral pneumonia	S5
InterviewWithCOVID19Patient	SSPTV News	March 30^th^, 2020	https://www.youtube.com/watch?v=dMbzMdqsWME	1	Female adult	S6
Like glass in your lungs Woman survives coronavirus after ICU experience 60 Minutes Australia	60 Minutes Australia	April 2^nd^, 2020	https://www.youtube.com/watch?v=-BINHWPmaKw	5	Female 30 something	S7= S11
Ohio man shares coronavirus experience $\vert $ ABC News	ABC News	March 16^th^, 2020	https://www.youtube.com/watch?v=hoTpRQtfnII	2	Male 56	S8 = S9
Warren coronavirus patient update	FOX 8 News Cleveland	March 19^th^, 2020	https://www.youtube.com/watch?v=VqCGPeGjKCQ	1	Male 56	S9 = S8
What it’s like having Covid-19 A Kiwi explains	RNZ	March 21^st^, 2020	https://www.youtube.com/watch?v=BzUwEYX5gaE	7	Female 40 something	S10
Woman in ICU warns of Covid-19 dangers ‘Don’t take any chances’	Guardian News	March 20^th^, 2020	https://www.youtube.com/watch?v=iFLSG-7K3Tc	6	Female	S11 = S7
Judy Gabriel Interview – April 23^rd^ 2020	CITL CKSA	April 23^rd^, 2020	https://www.youtube.com/watch?v=iPQWTmAVTV4	13	Female adult – self reported having bronchitis	S12 = S13
COVID-19 survivor still struggles with symptoms	Global New	April 25^th^, 2020	https://www.msn.com/en-ca/video/watch/covid-19-survivor-still-struggles-with-symptoms/vp-BB13axWe	3	Female adult, coughs after treatment hydroxychloroquine	S13 = S12

### B. Cough Segmentation

For each interview, cough events were manually segmented and assigned a label (C19_subjectNumber_coughNumber). Each file was then saved as a WAV file with a sampling rate of 44.1kHz. This resulted in 73 individual cough events. As these were cough events extracted from interviews, some contained background noise (i.e. speech or music). Additionally, some of the events were a mix between a throat clear and a cough event, which are labeled in the supplementary file. The supplementary file ‘coughDescriptions.txt’ is included with nine columns (Name; Duration (s); Number of Phases; Phase Notes; Competing Sources; Sex; Age; Live vs. Home (as described in section IV); Notes (including any self-reported underlying conditions)).

### C. Cough Phase Annotation

**FIGURE 1.**
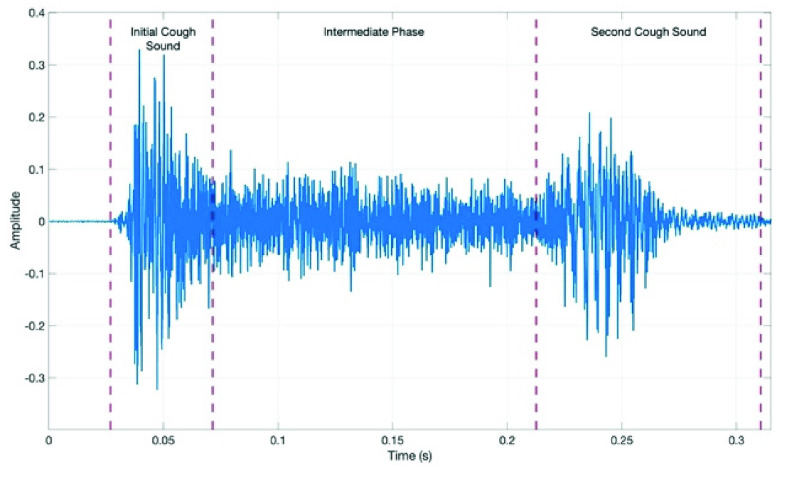
COVID-19 cough event from S1 with all phases present (filename = C19_1-1.wav).

There are three phases in a typical cough event [10], [23]. These phases are typically called the (1) initial cough sound, (2) intermediate phase, and (3) second cough sound [23]. Figure 1 presents an example COVID cough event with each phase identified. The underlying physiology behind these phases are outside the scope of this article, however a detailed description can be found in [10].

**FIGURE 2.**
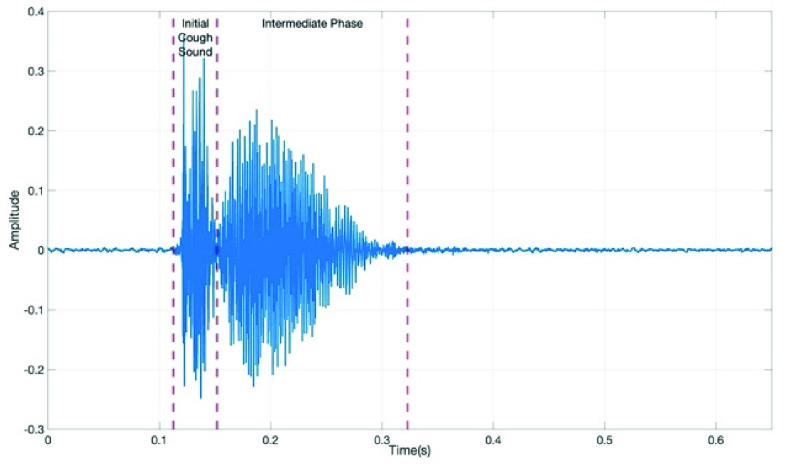
COVID-19 cough event from S1 with all phases present (filename = C19_1-1.wav).

Identification and labeling of these three phases in a cough event may highlight differences between types of cough events. For example, Chatrzarrin et *al.* used the power variations between the first and second phase as a feature for differentiating between wet and dry cough events. Furthermore, some cough events do not contain the third phase (Figure 2) [23]. The reduction or complete absence of this phase may also be a potential tool for differentiation.

For each cough event in NoCoCoDa, the two or three phases were annotated with time stamps. The annotations are included in a supplementary file ‘phaseAnnotations.txt’. There are five columns, cough event name, the start of each phase and the end of phase three if present. There is an assumption that consecutive phases occur immediately after one another. If there is no third phase present, the beginning of phase three was set at the end of phase two and the end of phase three was set to zero. In the case where the cough event included some component of a throat clear, only a single phase was identified by the bounds of ‘start of phase 1’ and ‘end of phase 1’, the remaining columns were set to zero. Table 3 contains a subset of some of the annotations.

**TABLE 3:** Subset of Phase Annotations

Cough	Start of Phase 1 (s)	Start of Phase 2 (s)	Start of Phase 3 (s)	End of Phase 3 (s)
C19_1–1.wav	0.058	0.042	0.163	0.447
C19_1–2.wav	0.126	0.354	0.470	0.534
$\vdots $	$\vdots $	$\vdots $	$\vdots $	$\vdots $
$\vdots $	$\vdots $	$\vdots $	$\vdots $	$\vdots $
C19_13–2.wav	0.114	0.206	0.428	0.490
C19_13–3.wav	0.045	0.107	0.256	0.364

## IV. COVID-19 Cough Characteristics

The cough associated with COVID-19 has been reported to be dry (non-productive) in the early phases of the disease [1], [6]. More recently however, there has been some reports indicating that the cough becomes more productive (wet) as the disease begins to affect the lungs [24].

From pathophysiological perspective, the presentation of both cough events can be explained. Once an individual is exposed to the COVID-19 virus it enters their system either through the mucus membrane or blood [25]. The virus then uses the angiotensin-converting enzyme 2 (ACE-2) receptor to enter the body cells [25].

The inhibition of the ACE-2 receptor due to the COVID-19 virus using it has a similar reaction in the body as individuals who take ACE-Inhibitors to treat heart conditions [26]. It has been reported that in a minority of patients who use these medications have a non-immune type-B hypersensitivity reaction that causes a chronic dry cough [26].

Once the virus enters a cell, it creates more viral proteins and viral RNA, which are packaged and released in order to attach to other cells with the ACE-2 receptors within the body [25]. Once the virus has affected lungs, the body will send neutrophils (white blood cells) to that area and release reactive oxygen species and cytokines [25]. This buildup of white blood cells is an inflammatory response, and depending on its severity can lead to the production of sputum, making it a productive cough [27]. Depending of the contents of the sputum, there could also be destruction of lung tissue (blood) and secondary infection of bacteria [28].

This possible transition from a dry to wet cough is notable among diseases with cough as a symptom. Therefore, cough progression among COVID-19 positive individuals may provide insight to diagnosis and disease progression as not everyone with COVID-19 will present with the more severe reaction that may be associated with productive cough events.

In this dataset, we have no definitive cough progression information. However, there are four subject interviews (5, 7, 8/9, 12/13) where an at home video (typically when they were hospitalized) of coughing was played in addition to live coughing events. Therefore, for these four subjects, there is at least one cough event from a more severe stage (home video) of the disease and at least one cough event from the recovery stage of the disease (live interview). These cough events were labeled as either ‘home’ or ‘live’ in the description file (column eight).

Unfortunately, we do not have access to cough events at the beginning of the disease progression, prior to the expression of more severe symptoms and/or hospitalizations. We therefore expect that at least some of the cough events present in this dataset will be more productive (wet) in nature due to the time at which they were recorded in the disease progression. Future data collection focusing on the progression from dry (early stages) to wet (later stages in severe cases) may lead to some findings that are unique for COVID-19.

## V. Cough Detection

The progression of cough is a clinical indicator of disease progression and deterioration [23]. Cough detection from audio recordings is a growing area of research that may be able to support medical professionals especially in the current period of physical distancing. Detection can be considered from two different perspectives.

Cough detection amongst other sounds (sometimes referred to as cough identification) can lead to a measure of cough frequency. Hoyos-Barceló *et.* al. implemented a cough event detection tool in real time using smartphone recordings. Their approach used a k-NN classifier with accuracies of 93% [29]. In [30] coughs were classified based on 8-dimentional number octonions. Classification of cough sounds vs. non-cough sounds using this method resulted in a sensitivity of 96% and a specificity of 98.4% [30]. Monge-Álvarez *et.* al. applied moment theory to detect cough events in noisy audio signals, which resulted in a sensitivity and specificity of about 90% [31].

The monitoring of human sounds (e.g. snoring) throughout the night is a related area of research. Vhaduri *et.* al. have investigated cough and snore detection in the presence of ambient room noise using smartphones. The k-NN classifier resulted in the highest accuracy (97%) when performing the binary classification between cough and snore events [32]. Voluntary cough detection against speech in a population of volunteers has also been investigated [33]. The authors used a classification tree to differentiate between cough and non-cough events resulting in a sensitivity of 100 % and a specificity of 95% [33].

The second cough detection perspective focuses on disease and or cough type differentiation. Here, it is assumed that cough events have previously been identified and further investigation into audio cough characteristics is needed for diagnosis and/or monitoring purposes. This area has received considerably less research and a fully automatic cough-based diagnosis tool has not been adopted in the medical field. However, Pramono *et.* al. has created a cough-based algorithm for automatic diagnosis of pertussis, more commonly known as whooping cough [34]. The focus of this algorithm is the differentiation between the classical whooping sound and all other events in a cough recording using a logistic regression model [34]. Among 38 patients, pertussis was diagnosed with 100% accuracy with zero false diagnosis. They also report that individual cough sounds were detected with a 92% accuracy [34].

Most of the aforementioned research focuses on a binary classification task between cough and non-cough related sounds (i.e. speech, ambient room noise and snore). A more complex problem is the differentiation between different cough types, which was evaluated in [34] to evaluate whooping sounds. Two very similar cough types that have clinical relevance are productive (wet) and non-productive (dry) cough types. The majority of research into productive vs. non-productive cough sounds has focused on feature evaluation [20], [35].

The COVID-19 cough has generally been reported as non-productive (dry). However, in severe cases the cough can progress and become more productive in nature. The following section describes the implementation of our previously created cough type detection tools to the NoCoCoDa, highlighting the fact that the majority of the cough events in the database were recorded at later stages of the disease. This is the first step in the differentiation between COVID-19 cough events and non-COVID-19 cough events, which is outside the scope of the current paper.

## VI. COVID-19 Cough Type Detection

As mentioned, the cough associated with COVID-19 can be dry during early stages and progress to become more productive as the disease progresses in a subset of severely affected individuals [24]. It is important to note that a single cough is rarely completely dry or completely wet. The lack of a clear separation leads to inconsistencies when coughs are being labeled by physicians. Therefore, it may be more accurate to say that a particular cough is more wet or dry in nature.

Our research group has previously investigated the differentiation of these two cough types [20]. We applied the methods described in Chatrzarrin et *al.* to the COVID-19 dataset in order to investigate if these coughs were identified as dry or wet cough events. The two features used were number of peaks present in the energy spectrum and the power ratio between the first two phases of each cough event [20]. We extended this work by creating a Linear Discriminant Analysis (LDA) classifier to differentiate between wet and dry cough events using our previously published data [20]. We then classified the COVID-19 events to detect if they were more wet or dry in nature.

**FIGURE 3.**
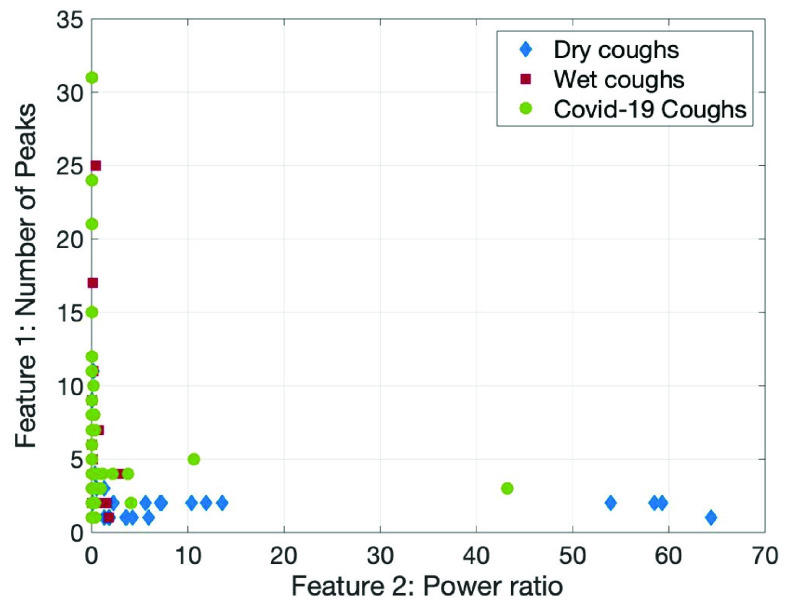
Power ratio vs. number of peaks feature comparison of COVID-19 coughs when compared to the wet and dry coughs events from [20].

We expected that some of the cough events, especially those recorded during hospitalization, were more likely to be wet given the more severe impact on the lungs. The results in Figure 3 indicate that the majority of cough events from the NoCoCoDa database have been identified as more wet like based on the low power ratio and higher number of peaks present in the energy spectrum. More specifically, after classification with the LDA, 77% of the COVID-19 cough events were identified as wet. This further supports our hypothesis that most of the cough events were from individuals with severe cases of the disease.

## VII. Current Issues with the Dataset

The NoCoCoDa contains COVID-19 cough events that were obtained from online sources. Though using this dataset may be very beneficial for early research, the end goal would be to collect a more controlled dataset of COVID-19 cough events and apply the early methods designed using NoCoCoDa to the new data.

We have identified some of the issues associated with the current iteration of this dataset, which are as follows.

### A. Inconsistent Recording Methods

Recording methods are unknown in all cases. Interviewees are presented via online video chat, which may introduce additional recording errors. At home videos of patients during severe stages of the disease are also presented and recording methods are also unknown.

### B. Competing Sources

As the cough events were taken from interviews, there are periods where multiple individuals are speaking. Sometimes cough events are present with speech from the interviewer. There are also a few cases, where music was overlaid over speech or an at home video of cough events.

### C. Self Report Labeling Methods

All of the cough events in NoCoCoDa are labeled based on the assumption that they are indeed interviews with COVID-19 positive individuals. Additionally, all interviews presented were from news sources and assumed that they have previously done their own due diligence.

There are a few recordings where the interviewee disclosed that they have underlying conditions. Those labels have also been included here, but again are based on the assumption that interviewees are correct and complete.

Though most of the live interviews indicated that they were done after the interviewee has recovered to some extent, we do not know how severe their lung involvement was for either the live or home cough events. Furthermore, the disease stage for the home videos, often recorded while interviewees were in hospital, is also unknown.

### D. Small and Inconsistent Sample Size

NoCoCoDa is restricted by the available resources, meaning that there have only been 11 subjects identified. Furthermore, the number of cough events per subject varies considerably as every cough event was a reflex cough.

## VIII. Conclusion

Here we have presented the NoCoCoDa of 73 COVID-19 reflex cough events. The data was collected by identifying interviews with COVID-19 positive individuals and manually segmenting cough events. Furthermore, we have described the supplementary files that include cough event descriptions and cough phase annotations.

COVID-19 coughs have a notable pathophysiology, specifically how they may transition from a dry cough (one of the most common symptoms) to a more productive (wet) cough in severe cases of the disease. We highlighted this by applying our previous feature comparison method aimed to differentiate between wet and dry cough events. The majority (77%) of COVID-19 cough events have been identified as being more wet in nature, indicating that the interviewees were probably in the subset of patients who had more severe reactions and recordings were done during that time.

Finally, we have discussed some of the short falls of this dataset including; inconstant recording methods, competing sources, self-report labeling methods and small and inconsistent sample sizes. We present this dataset as a preliminary tool to create possible COVID-19 cough identification and tracking tools that may later be applied to a more through dataset. The long-term goal is to implement tools derived using NoCoCoDa in unobtrusive monitoring environments (residential, public and continuing care homes) to monitor the frequency, severity and cough characteristics in order to aid contract tracing of disease and provide medical professionals with more accurate information for their clinical assessments.
